# Long COVID in pediatrics—epidemiology, diagnosis, and management

**DOI:** 10.1007/s00431-023-05360-y

**Published:** 2024-01-27

**Authors:** Nicole Toepfner, Folke Brinkmann, Silvia Augustin, Silvia Stojanov, Uta Behrends

**Affiliations:** 1 Department of Pediatrics, Faculty of Medicine and University Hospital Carl Gustav Carus, Technische Universität Dresden, Dresden, Germany; 2 Division of Pediatric Pulmonology and Allergology, University Children’s Hospital, Airway Research Center North (ARCN), German Center for Lung Research (DZL), Luebeck, Germany; 3 MRI Chronic Fatigue Center for Young People, Pediatrics, Children’s Hospital, Technical University Munich and Munich Municipal Hospital, Munich, Germany; 4 MRI Chronic Fatigue Center for Young People, Child and Adolescent Psychosomatics, Children’s Hospital, Technical University Munich and Munich Municipal Hospital, Munich, Germany

**Keywords:** Post-COVID syndrome (PCS), Post-COVID-19 condition (PCC), Long COVID, Post-acute sequelae of COVID-19 (PASC), Myalgic encephalomyelitis/chronic fatigue syndrome (ME/CFS), Post-exertional malaise (PEM)

## Abstract

This review summarizes current knowledge on post-acute sequelae of COVID-19 (PASC) and post-COVID-19 condition (PCC) in children and adolescents. A literature review was performed to synthesize information from clinical studies, expert opinions, and guidelines. PASC also termed Long COVID — at any age comprise a plethora of unspecific symptoms present later than 4 weeks after confirmed or probable infection with severe respiratory syndrome corona virus type 2 (SARS-CoV-2), without another medical explanation. PCC in children and adolescents was defined by the WHO as PASC occurring within 3 months of acute coronavirus disease 2019 (COVID-19), lasting at least 2 months, and limiting daily activities. Pediatric PASC mostly manifest after mild courses of COVID-19 and in the majority of cases remit after few months. However, symptoms can last for more than 1 year and may result in significant disability. Frequent symptoms include fatigue, exertion intolerance, and anxiety. Some patients present with postural tachycardia syndrome (PoTS), and a small number of cases fulfill the clinical criteria of myalgic encephalomyelitis/chronic fatigue syndrome (ME/CFS). To date, no diagnostic marker has been established, and differential diagnostics remains challenging. Therapeutic approaches include appropriate self-management as well as the palliation of symptoms by non-pharmaceutical and pharmaceutical strategies.

*Conclusion*: PASC in pediatrics present with heterogenous severity and duration. A stepped, interdisciplinary, and individualized approach is essential for appropriate clinical management. Current health care structures have to be adapted, and research was extended to meet the medical and psychosocial needs of young people with PASC or similar conditions.

**Table:** 

**What is Known:** *• Post-acute sequelae of coronavirus 2019 (COVID-19) (PASC) — also termed Long COVID — in children and adolescents can lead to activity limitation and reduced quality of life.* *• PASC belongs to a large group of similar post-acute infection syndromes (PAIS). Specific biomarkers and causal treatment options are not yet available.*	
**What is New:** *• In February 2023, a case definition for post COVID-19 condition (PCC) in children and adolescents was provided by the World Health Organization (WHO), indicating PASC with duration of at least 2 months and limitation of daily activities. PCC can present as myalgic encephalomyelitis/chronic fatigue syndrome (ME/CFS).* *• Interdisciplinary collaborations are necessary and have been established worldwide to offer harmonized, multimodal approaches to diagnosis and management of PASC/PCC in children and adolescents.*

**What is Known:**

*• Post-acute sequelae of coronavirus 2019 (COVID-19) (PASC) — also termed Long COVID — in children and adolescents can lead to activity limitation and reduced quality of life.*

*• PASC belongs to a large group of similar post-acute infection syndromes (PAIS). Specific biomarkers and causal treatment options are not yet available.*

**What is New:**

*• In February 2023, a case definition for post COVID-19 condition (PCC) in children and adolescents was provided by the World Health Organization (WHO), indicating PASC with duration of at least 2 months and limitation of daily activities. PCC can present as myalgic encephalomyelitis/chronic fatigue syndrome (ME/CFS).*

*• Interdisciplinary collaborations are necessary and have been established worldwide to offer harmonized, multimodal approaches to diagnosis and management of PASC/PCC in children and adolescents.*

## Introduction

Shortly after the onset of the pandemic, the term Long COVID had been introduced by people experiencing a broad variety of persisting symptoms following the infection with severe acute respiratory syndrome coronavirus type 2 (SARS-CoV-2) [1]. Long-term symptoms were described after any clinical course of the initial SARS-CoV-2 infection and at any age, with impairment of many people’s daily activities and health-related quality of life (HRQoL) [2]. The umbrella terms Long COVID and post-acute sequelae of COVID-19 (PASC) are mostly being used for SARS-CoV-2-associated symptoms later than 4 weeks [3], and “Post-COVID-19 Syndrome” (PCS) for symptoms later than 12 weeks after the initial infection [4, 5]. A post-COVID-19 condition (PCC) was defined by the WHO in October 2021 as the continuation or development of new symptoms 3 months after the initial SARS-CoV-2 infection, with these symptoms lasting for at least 2 months and no other explanation [6]. To indicate PCC, the assign code U09.9! was added to the 10th Revision of International Classification of Diseases internationally (ICD-10) [7, 8]. However, no PCC case definition was provided for children until February 2023 [6], and evidence from pediatric research still is not conclusive regarding many features of PASC and PCC [9, 10]. This is due to the large heterogeneity of study designs, the paucity of studies including uninfected control groups, and the limited representativity of studies from distinct countries due to different courses of the pandemic and different strategies of its management worldwide [9, 10]. This review aims at summarizing current clinical perspectives on PASC and similar disorders.

## Materials and methods

A literature search was conducted in PubMed between March 20, 2023, and June 20, 2023, with search terms combined by Boolean operators (AND, OR) and truncated search terms according to the PubMed User Guide. PubMed’s Automatic Term Mapping was applied as well as the following MESH terms used: (Long COVID) OR (Post-COVID) OR (Post COVID) OR (post-acute AND COVID) OR (post-acute AND SARS*) OR (sequelae AND COVID) OR (sequelae AND SARS*) OR (ME/CFS) OR (chronic fatigue syndrome) AND (pediatr*) OR (paediatr*) OR (child*) OR (kid*) OR (infant*) OR (toddler*) OR (pre-schooler*) OR (preschooler*) OR (adolescen*) OR (youth*) OR (teen*). Additional literature was added based on non-systematical online search in PubMed and on preprint servers as well es in the ClinicalTrials.gov Protocol Registration and Results System. To narrow the search results, the primary review inclusion criteria were applied using the following filters: species = human, language = English, and patients below 18 years of age. Evidence from studies with adult patients was discussed and added when appropriate. The final decision to include studies into the review was consented by all authors.

## Results

### Definition

**Fig. 1:**
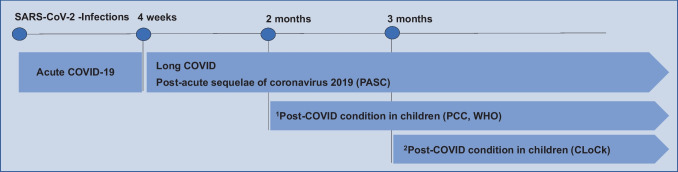
Definition of long-term sequelae of coronavirus disease 2019 (COVID-19) in children

In addition to earlier definitions for PASC and PCS at any age [11, 12], a pediatric research definition was suggested for Long COVID/PCC by a modified Delphi process in 2022 [13]. In February 2023, a clinical case definition of a PCC in children was provided by the WHO [14]. According to this definition, PCC “occurs in individuals with a history of confirmed or probable SARS-CoV-2 infection, when experiencing symptoms lasting at least two months which initially occurred within three months of acute COVID” (Fig. 1). The WHO stated that “symptoms generally have an impact on everyday functioning such as changes in eating habits, physical activity, behavior, academic performance, social functions (..) and developmental milestones” and, moreover, “may be new onset following initial recovery from an acute COVID-19 episode or persist from the initial illness” and “may also fluctuate or relapse over time.” Whether a SARS-CoV-2-triggered exacerbation or aggravation of pre-existing morbidities (e.g., bronchial asthma, migraine, and depression) should be excluded from the PCC definition at any age is still a matter of debate [15].

In addition to earlier definitions for long-term sequelae of coronavirus 2019 (COVID-19), a pediatric definition for a post-COVID condition (PCC) was suggested by a modified Delphi process within the CloCk consortium (CLoCk) in July 2022 for research purposes [13] and by the World Health Organization (WHO) in February 2023 for clinical use [14]. Definitions were as follows: ^1^Post COVID-19 condition (PCC, WHO), “in children and adolescents occurs in individuals with a history of confirmed or probable SARS-CoV-2 infection, when experiencing symptoms lasting at least 2 months which initially occurred within 3 months of acute COVID-19... Symptoms generally have an impact on everyday functioning... Symptoms may be new onset following initial recovery from an acute COVID-19 episode or persist from the initial illness. They may also fluctuate or relapse over time. Workup may reveal additional diagnoses, but this does not exclude the diagnosis of post COVID-19 condition. This can be applied to children of all ages, with age-specific symptoms and impact on everyday function taken into consideration”. (2/2023) ^2^Post-COVID condition in children (CLoCk), “occurs in young people with a history of confirmed SARS-CoV-2 infection, with at least one persisting physical symptom for a minimum duration of 12 weeks after initial testing that cannot be explained by an alternative diagnosis. The symptoms have an impact on everyday functioning, may continue or develop after COVID infection, and may fluctuate or relapse over time. The positive COVID-19 test referred to in this definition can be a lateral flow antigen test, a PCR test or an antibody test”. (7/2022)

### Clinical features

PASC present with a variety of unspecific symptoms [14] and significant inter-individual heterogeneity at any age [16]. Fatigue, altered smell or anosmia, and anxiety were pointed out by the WHO as symptoms most strongly associated with PCC in children and adolescents, with 21 additional symptoms listed, including chest pain, cognitive difficulties, cough, diarrhea, dizziness, dyspnea, earache/ringing in ears, fever, headache, insomnia, joint pain or swelling, light sensitivity, loss of appetite, mood swings, myalgia, nausea, palpitations, postural symptoms, rash, stomach ache, and sore eyes or throat [14, 17]. Like in adults, “brain fog” which comprises various cognitive impairment sequelae is reported by adolescents and children [17, 18]. Fatigue was identified as one of the most frequent symptoms of PASC at any age in many studies [19, 20] and together with “post-exertional malaise” (PEM) represents the cardinal symptoms of ME/CFS (ICD-10 G93.3) [21–23]. PEM is frequent in PASC as well and was termed post-exertional symptom exacerbation (PESE) or post-exertional neuroimmune exhaustion (PENE) in some studies [24–27]. It is defined as a worsening of symptoms after daily activities that were well-tolerated before, often manifests only 12 to 48 h after activity, and can last for days or even weeks [28].

ME/CFS was documented as severe subtype of PCC in adults [29, 30] and was recently reported by our pediatric group in patients with PCC younger than 18 years [31]. ME/CFS is associated with a HRQoL lower than in other chronic diseases, and significantly impairs social participation in most cases [21, 22, 32]. The clinical features of PASC resemble those of other post-acute infection syndromes (PAIS). PAIS had been described for decades at any age, including ME/CFS [33–37]. PAIS can occur after infection with viruses, bacteria, and protozoa, with EBV representing a prominent trigger in adolescents and children [38, 39]. Importantly, post-acute vaccination syndromes (PAVS) with similar clinical features have recently been reported in adults, adolescents, and children after COVID-vaccination (post-COVIDvac/Post-Vac-syndrome) [40, 41].

### Epidemiology

Since the emergence of the SARS-CoV-2 variant omicron (B.1.1.529), the COVID-19 pandemic has gradually evolved into an endemic situation. Meanwhile, most adolescents and children had one or more contacts with SARS-CoV-2 and/or its spike antigen included in COVID-19 vaccines [42, 43]. With the increasing probability of SARS-CoV-2 infection in history, reduced SARS-CoV-2 testing worldwide, and non-persistence of SARS-CoV-2 antibodies in many individuals, a clear attribution of persisting or new symptoms to a previous SARS-CoV-2 infection is not possible in many cases. However, the lack of a documented specific infection is frequent in PAIS, and the diagnosis, in these cases, has to be based on a probable initial infection. This diagnostic challenge is well known in pediatrics from PAIS after Epstein-Barr virus (EBV)-associated infectious mononucleosis [17, 39, 44].

The prevalences of PASC and PCC as well as subgroups thereof in the pediatric age group are still largely unknown. PASC was reported with 25% in a large pediatric meta-analysis [9]. An umbrella review of PCC in initially non-hospitalized children found a prevalence of 2.0–3.5% [45]. The pooled prevalence of PCC symptoms in studies with a supposedly SARS-CoV-2-negative control group was lower than in uncontrolled studies [46]. Controlled studies reported a range from less than 1% to more than 40%, with less than 5% in most studies. A final conclusion on prevalence is difficult due to the heterogeneity regarding definitions as well as addressed cohorts (e.g., populations-based, hospital-based) and investigated symptoms. Data were collected from self-reports in many studies, and controls were selected by different measures [9, 10, 45–47].

### Pathogenesis and risk factors

Several pathogenic mechanisms have been suggested for PASC and other PAIS. These include persistence of virus and/or viral components, virus-induced tissue damage, endothelial dysfunction, coagulopathy, autonomic dysfunction, chronic inflammation, and autoimmunity [35, 36, 47–51]. The risk of developing PASC is two to fourfold higher after infection with a pre-omicron variant [52, 53], and COVID-19 vaccination was shown to reduce the risk of PASC, depending on the triggering virus variant [53–56]. Moreover, higher age, female gender, and pre-existing health issues were suggested to increase the risk in the pediatric population [9, 46, 47, 57, 58]. A score was suggested for risk calculation in pediatrics, including SARS-CoV-2 status, number of symptoms at testing, sex, age, ethnicity, physical and mental health, loneliness, and four EQ-5D-Y scale items (problems looking after self, doing usual activities, having pain, and feeling worried/sad) before testing [59]. Immunological genetic variants are addressed as candidate risk factors in ongoing studies [60]. However, risk factors for severe compared to mild and moderate PASC subtypes are largely unknown.

### Diagnostics

No biomarker is available yet for the diagnosis of PASC which therefore has to be differentiated from other medical conditions that may elicit similar symptoms, including other PAIS. Of 110 pediatric patients with suspected PASC admitted to a specialized outpatient clinic, only 29% received a final PASC diagnosis while 47% were diagnosed with alternative somatic/mental diseases, and 23% with diagnosis unclarified [61]. Differential diagnoses include a broad variety of somatic (e.g., pulmonary, cardiovascular, neurological, rheumatic, oncological, gastrointestinal, and metabolic) and psychiatric (e.g., depression, anxiety, attention deficit (hyperactivity), somatization, conversion, and eating) disorders as well as mental distress due to social distancing or loss of relatives during the pandemic (“long-lockdown,” “post-pandemic-syndrome”). Racine et al. reported almost a doubling of the global prevalence of depressive and anxiety symptoms in children and adolescents during the pandemic as compared to before [62]. In Germany, although mental health of the youth improved in year three of the pandemic, it is still lower than pre-pandemic [63]. Accordingly, many studies revealed high loads of unspecific symptoms in both SARS-COV-2-infected cases and SARS-COV-2-non-infected controls. For example, 1560 students recruited from Dresden schools with or without detectable anti-SARS-CoV-2 antibodies in 2020/21 showed similar rates of concentration problems, listlessness, fatigue, and mood swings (40–80%) [64]. The LongCOVIDKidsDK study found 46% and 41% adolescents with or without a positive SARS-CoV-2 test in history reporting PCC-like symptoms, respectively [65]. In the CLoCk Study, 24.5% and 17.8% of initially SARS-CoV-2 test-positive and SARS-CoV-2 test-negative children and adolescents had PCC-like symptoms at 6 months [66]. Furthermore, somatic and especially psychiatric comorbidities have to be carefully taken into account. Thus, PASC/PCC-like symptoms are challenging both clinical diagnostics as well as epidemiological studies, especially in children.

Therefore, in order to ensure a thorough diagnostic assessment, it should be aimed to evaluate patients simultaneously by a pediatrician and child and adolescent psychiatrist or psychologist. Algorithms have been published for adults, adolescents, and children with PASC to allow for a stepped and individualized diagnostic approach [4, 5, 18]. Diagnostics include a thorough medical and psychosocial history, usually in form of a semi-structured qualitative interview, a comprehensive physical exam and psychological assessment, as well as routine blood analyses, supplemented by function tests, neuropsychological testing, and/or imaging, depending on the individual symptoms. Basic blood analyses should address differential cell count, C-reactive protein (CRP), liver, renal, and thyroid function, glucose, as well as anti-nuclear and anti-transglutaminase antibodies. Endocrinopathies can manifest in the context of COVID-19 and therefore need special attention [67, 68]. Basic function tests comprise Holter electrocardiogram, pulmonary function test, passive standing test [69], 6-min walk test, and/or 1-min sit-to-stand test. The passive standing test (or head-up tilt table test) aims at detecting postural tachycardia syndrome (PoTS) in patients with a history of orthostatic symptoms. PoTS is defined by a sustained heart rate ≥ 120 beats per minute (bpm) and/or a sustained heart rate increase by ≥ 40 bpm within 10 min standing compared to supine for individuals ≤ 19 years [4, 5, 47, 70]. Supplementary tests might include an electroencephalogram, cardiac and/or lung ultrasound, chest X-ray, computer tomography, magnet resonance imaging (MRI), cardiopulmonary exercise testing (CPET) and/or ventilation–perfusion single-photon emission computed tomography (V/Q SPECT), and neurocognitive testing. Both SPECT/CT and CPET suggested pulmonary circulation dysfunction in a 13-year-old child [71]. However, low-field-strength MRI showed persistent pulmonary dysfunction in both pediatric patients who recovered from COVID-19 and those with PASC [72]. With regard to neurocognitive testing so far, there is no PASC specific test battery available, and pediatric data mainly lacking. It might be challenging to select appropriate diagnostic measures, and patients with severe PEM might not tolerate the full routine work-up.

Early identification of PEM is important to both stratifying diagnostics and management. In order to detect PEM, the brief DePaul Symptom Questionnaire (DSQ) was developed which addresses the severity, frequency, and duration of PEM symptoms at any age. It correctly categorized adult patients with ME/CFS 81.7% of the time, while incorrectly categorizing multiple sclerosis (MS) and post-polio syndrome as ME/CFS only 16.6% of the time [73]. We have used the DSQ-PEM together with our novel Munich Berlin Symptom Questionnaire (MBSQ) to identify PEM and ME/CFS in children, adolescents, and adults with PCC, respectively [31]. Semi-structured qualitative interviews before and after CPET in adults indicated that patients had unique PEM experiences, with differences regarding onset, severity, trajectory over time, and most bothersome symptoms [74]. However, little is known on PCC-PEM in children and adolescents, and CPET is not applicable to routine diagnostics of people with probable PEM due to the risk of worsening the symptoms.

### Management

No specific causal treatment was established yet for PAIS, including PASC and ME/CFS. However, all patients should be offered appropriate holistic care, including non-pharmaceutical and pharmaceutical approaches as individually needed. The most bothersome symptoms should be prioritized, and multi-modal, multi-professional strategies are developed for those with complex and/or severe symptoms [4, 5, 18, 47, 70, 75].

As a first step, disease-specific education should be provided to patients and parents about current knowledge, remaining uncertainties, and patient advocacy groups. An introduction to self-management strategies [4, 5, 18, 47, 70, 75] should address relaxation techniques, deep breathing exercises, sleep hygiene, gentle muscle training if tolerated, coping strategies, as well as a the 3P energy conservation strategy (prioritizing, planning, and pacing) for patients with PEM [4, 5, 75]. The pacing means to be as active as possible within the individual limits, without inducing PEM [76, 77].

Depending on the individual needs, non-pharmaceutical options of care also include physiotherapy, respiratory therapy, psychotherapy, cognitive training, occupational therapy, and transcutaneous electrical nerve stimulation (TENS), as well as the prescription of medical aids (e.g., compression stockings, wheel chair), appropriate social support (e.g., certificates for schools, applying for an appropriate grade of disability), and home care in severe cases [4, 5, 47, 70].

Pharmaceutical treatment should follow current guidelines (e.g., for pain, sleeping disorders, migraine), and deficits of vitamins or minerals should be supplemented [4, 5, 18, 47, 70]. However, off-label use of drugs which are approved for other indications might help in some cases (e.g., for PoTS or “brain fog”). Ideally this would occur after consulting specialists with pediatric expertise in treating PAIS and/or ME/CFS, recognizing that these resources might not be available in all locations. Patients with bronchial hyperresponsiveness may benefit from inhaled corticosteroids [35]. Orthostatic intolerance may improve with adequate salt and fluid intake, compression stockings, gentle exercises, as well as additional measures to modulate heart rate (e.g., ivabradine) and/or stabilize blood pressure (e.g., midodrine). Up to now, anticoagulation without clear evidence of a coagulation disorder is not indicated [36].

In adults, several randomized controlled trials (RCT) are investigating pharmaceutical approaches to PCC, including antiviral drugs as well as immunomodulatory, neuromodulatory, and vasoactive substances [78]. A prominent approach is the depletion of pathogenic autoantibodies, e.g., by plasma exchange, immunoadsorption, or infusion of the aptamer BC007 [79]. Hyperbaric oxygen therapy (HBOT) was shown to improve neurocognitive function in a phase II RCT for PCC [80] and will be further investigated within our National Clinical Study Group on PCC and ME/CFS (NKSG) [78]. We are not aware of any published results from interventional trials addressing PSC in children or adolescents. The website ClinicalTrials.gov lists an ongoing phase 2a randomized, double-blind, placebo-controlled clinical trial evaluating larazotide (AT1001) [81] and a double-blind, controlled trial investigating vitamin D [82].

### Prognosis

For the majority of children and adolescents with PASC, the prognosis is favorable, and symptoms decline within 6 months [83, 84]. Accordingly, several pre-pandemic studies indicated that a significant number of children and adolescents with PAIS, including ME/CFS following primary EBV infection, recovered [32, 38, 44]. A pediatric long-term follow-up study of ME/CFS reported that “paying attention to social learning needs and educational support have been identified by young people as being of similar importance to medical management” [32].

### Limited social participation and school absenteeism

Many students with PAIS, including PASC and ME/CFS, have to limit or omit sports and/or after-school activities, and some might not be able to attend school full- or part-time. Pre-pandemic ME/CFS was identified as the most common cause of long-term school absence [85–89]. However, the effect of PASC on school performance is not well studied yet [90]. Remarkably, a recent survey from Germany reported a 38% increase of general school absenteeism compared to pre-pandemic years [38], in Belgium school absenteeism in secondary education also increased by 56% between 2020/2021 and 2021/2022 [91]. Early detection of school absenteeism, education of school staff about PAIS and PEM, as well as appropriate diagnostics to differentiate between somatic, psychiatric, and social causes seem increasingly important.

### Stigmatization and disbelieve

Many patients with severe PAIS still are facing inadequate medical support, stigmatization, and disbelieve [42, 89, 90, 92, 93]. Parents of patients with PASC reported on “medical gaslighting” or “trivialization” of their concerns via social media [94] and asked for treatment with “compassion” [95]. A survey with adult PACS patients indicated that they were “encountering medical professionals who dismissed their experience, leading to lengthy diagnostic odysseys and lack of treatment options” [96]. Accordingly, current surveys indicate that patients with pre-pandemic ME/CFS and patients with PASC seek mutual support [97]. Parent advocacy groups such as “Long COVID KIDS” [98] and others [99, 100] might be helpful to families burdened by PAIS in children and adolescents.

## Discussion

### Stepped approaches to individualized, holistic care

PAIS, including PASC and ME/CFS, are complex disorders with non-specific symptoms affording appropriate differential diagnosis and multi-modal, multi-professional treatment in many cases. Primary physicians may initiate first steps to diagnosis, with following steps depending on the severity and duration of the individual illness. Cases with significant loss of participation should be prioritized for admission to specialists. First PCC and ME/CFS competence centers have been implemented, providing complex patient care as well as the transfer of knowledge and research. At all steps of patient care, a holistic, individualized approach is recommended, and care should be offered to patients with PAIS and PAVS [4, 5, 47, 70]. However, so far, the number of specialized institutions in Germany and Europe does not meet the urgent needs, and budgets provided by health insurances currently do not cover expenses at any level of sector-spanning, stepped medical care.

### Challenges of post-exertional malaise

Exertion intolerance with PEM is a challenging current health care concept. Affected patients might not be able to tolerate routine diagnostics and be unable to follow all therapeutic recommendations. Medical procedures have to be adapted to allow for breaks, to shorten distances, to reduce light and noises, and to prioritize the most bothering problems. This is true for the setting of the primary physician as well as for secondary and tertiary health care institutions, including rehabilitation facilities. Some patients with PEM or ME/CFS are not mobile enough to visit any medical institution and need multi-professional, multi-modal home treatment, supported by telemedical advice from specialists [4, 5, 18]. However, options for adequate multi-professional rehabilitation, home care, and telemedicine are not available yet as needed, and strategies have to be developed to protect affected patients from stigmatization and inadequate treatment. Because orthostatic stress can aggravate symptoms of ME/CFS and PASC/PCC, it is reasonable to propose that treating orthostatic stress has the potential to help with managing PEM, although more research on this topic is needed [101–106].

### Core outcome sets and future research

Clinical features and pathomechanisms of PAIS are increasingly addressed in pediatric studies, including ours [107–111]. However, a lack of harmonization limits the comparability worldwide. A library of common data elements was implemented before the pandemic in the USA to streamline research on ME/CFS [112], and a core outcome set (COS) for PCC in adults and children were recently agreed upon [113] and initiated [114], respectively. International collaborations on PAIS and ME/CFS are urgently needed to fill the gap of pediatric data and thereby pave the way to optimal patient care and prevention.

## Data Availability

As all data included in this review are already available via the cited references, a special statement on data availability is not applicable for this article.

## Abbreviations

COVID-19: Coronavirus disease 2019
CRP: C-reactive protein
CT: Computed tomography
CPET: Cardiopulmonary exercise testing
DSQ: DePaul Symptom Questionnaire
EBV: Epstein-Barr virus
HBOT: Hyperbaric oxygen therapy
HRQoL: Health-related quality of life
ICD-10-GM: 10Th Revision of the International Classification of Diseases–German Modifications
MBSQ: Munich Berlin Symptom Questionnaire
ME/CFS: Myalgic encephalomyelitis/chronic fatigue syndrome
MIS-C: Multisystem inflammatory syndrome in children
MRI: Magnetic resonance imaging
MS: Multiple sclerosis
NKSG: National Clinical Study Group
PACS: Post-acute COVID-19 sequelae
PAVS: Post-acute vaccination syndromes
PCC: Post-COVID-19 condition
PCS: Post-COVID syndrome
PEM: Post-exertional malaise
PENE: Post-exertional neuroimmune exhaustion
PESE: Post-exertional symptom exacerbation
PICS: Post-intensive care syndrome
PIMS: Pediatric inflammatory multisystem syndrome
PoTS: Postural tachycardia syndrome
RCT: Randomized controlled trial
SARS-CoV-2: Severe acute respiratory syndrome coronavirus type 2
SPC: Social Pediatrics Center
SPECT/CT: Single-photon emission computed tomography
TENS: Transcutaneous electrical nerve stimulation
V/Q SPECT: Ventilation–perfusion single photon emission computed tomography
WHO: World Health Organization
